# Effects of Ambient Temperature and Relative Humidity on Subsurface Defect Detection in Concrete Structures by Active Thermal Imaging

**DOI:** 10.3390/s17081718

**Published:** 2017-07-26

**Authors:** Quang Huy Tran, Dongyeob Han, Choonghyun Kang, Achintya Haldar, Jungwon Huh

**Affiliations:** 1 Department of Civil and Environmental Engineering, Chonnam National University, Yeosu, Chonnam 59626, Korea; 157042@live.jnu.ac.kr (Q.H.T.); hozilla@chonnam.ac.kr (D.H.); kangsmile@chonnam.ac.kr (C.K.); 2 Department of Civil Engineering and Engineering Mechanics, University of Arizona, Tucson, AZ 85721, USA; haldar@u.arizona.edu

**Keywords:** concrete deterioration, active thermal imaging, ambient temperature, relative humidity, infrared thermography, nondestructive testing

## Abstract

Active thermal imaging is an effective nondestructive technique in the structural health monitoring field, especially for concrete structures not exposed directly to the sun. However, the impact of meteorological factors on the testing results is considerable and should be studied in detail. In this study, the impulse thermography technique with halogen lamps heat sources is used to detect defects in concrete structural components that are not exposed directly to sunlight and not significantly affected by the wind, such as interior bridge box-girders and buildings. To consider the effect of environment, ambient temperature and relative humidity, these factors are investigated in twelve cases of testing on a concrete slab in the laboratory, to minimize the influence of wind. The results showed that the absolute contrast between the defective and sound areas becomes more apparent with an increase of ambient temperature, and it increases at a faster rate with large and shallow delaminations than small and deep delaminations. In addition, the absolute contrast of delamination near the surface might be greater under a highly humid atmosphere. This study indicated that the results obtained from the active thermography technique will be more apparent if the inspection is conducted on a day with high ambient temperature and humidity.

## 1. Introduction

**Figure 1:**
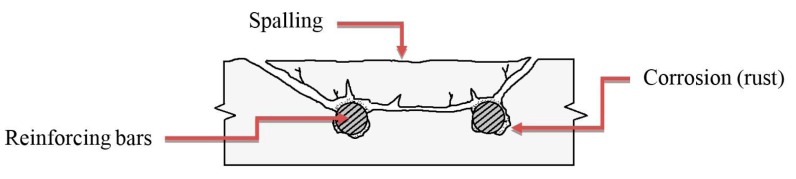
Crack, delamination, and spalling caused by expansion of corroded steel.

Concrete deterioration such as cracking, delamination, and spalling is often the result of a combination of factors such as corrosion of embedded metals, accumulative effect of freeze-thaw successive cycles, chemical attack, alkali-aggregate reactivity, or cracking due to shrinkage [1]. On the other hand, abrasion or overloading on bridge decks and highway pavements due to traffic operations can lead to cracking on the structure’s surface. Of these factors, the corrosion of embedded reinforcing steel in a concrete structure is the most common factor [2]. As a result, delaminations are initially developed at or near the level of the reinforcing bars because rust occupies a greater volume than steel, creating unexpected tensile stresses in the concrete [1,3]. Cracking and delaminations might be invisible upon visual inspection until the deterioration develops into a spalling condition in its later stages, as shown in Figure 1. This raises safety concerns, especially for the traffic passing under overpass bridges.

To address this problem, thermal imaging/infrared thermography (IRT) has proved to be an effective inspection tool in detecting subsurface deteriorations among other nondestructive techniques (NDT) such as impact-echo, coin tapping, ultrasonic surface waves or ground penetrating radar. One of the most significant advantages of the IRT technique is its short detection time compared to other techniques, and it is possible to qualitatively evaluate the area and depth of a subsurface delamination [4,5,6,7]. However, quantifying the delamination depth appears to be a considerable challenge, and only a few researchers have attempted to evaluate the depth of delamination using active IRT to inspect concrete structures. In addition, while changes in environmental conditions have a significant effect on experimental results, the ambient temperature, relative humidity, clouds, wind speed, and solar radiation should be considered during field testing [8]. Currently, previous studies have just focused on the passive IRT technique when considering the environmental influence on the concrete detection, as mentioned in detail in Section 2, while the impact of the environment on the active IRT technique is still an open question. 

The aim of this study is to survey concrete structural components that are not exposed directly to the sunlight and are less affected by the wind, such as inside a bridge box-girder, areas between the T or I-girder and diaphragms, and other structural components in the interior of buildings and factories, and so on. The impulse thermography technique, one of the active IRT techniques, is applied with six 500-watt halogen lamps working as external heat sources to transfer heat flux into the concrete specimen. The impulse thermography technique is considered throughout twelve cases of different ambient temperature and relative humidity conditions on a concrete slab in the laboratory. Our purpose in this study is to describe several important characteristics of heat transfer that affect the inspection results of concrete with different environmental conditions, and propose a preliminary selection of a suitable weather conditions for site inspection when using the active thermography technique.

## 2. Related Work

An early application of IRT in the civil engineering field related to health monitoring of concrete structures was performed by Clemena et al. in 1978 [9]. Their research found that more severely delaminated regions induce a stronger thermal contrast and delaminated concrete was warmer than non-delaminated concrete when directly exposed to sunlight. Other studies were conducted by Cielo et al. in 1987 [10] and by Maldague in 1993 [11], focusing on delamination depth. In these studies, they observed changes in the surface temperature of delaminated layers and the observation time to estimate the depth of delamination. In 2013, Vaghefi [5] tested four concrete slabs with delaminations placed at various depths to confirm the technique proposed by Maldague [11]. In 2008, Pollock et al. [4] conducted active thermal imaging inspections on six concrete specimens with embedded artificial air voids and post-tensioning ducts (PT-ducts) to simulate post-tensioned box girder bridges. It was shown that the PT-ducts and simulated voids in the 20 cm thick specimens were more apparent than in the 30 cm thick specimens. Also, PT-ducts were much clearer and more visible in the thermal images of the thinner specimens. However, it was found that two factors need to be considered: accessibility (whereby the heat source needs to be placed inside the box girder) and cost (whereby the technique is time-consuming when testing for relatively deep air voids).

For assessing the influence of environmental conditions on the passive IRT results, in 2009 Washer et al. [12] developed guidelines on how to use the technology and where it could be applied by characterizing the environmental conditions (i.e., solar loading, diurnal temperature variations, wind speed, and relative humidity) necessary for effective thermography in the field. In Washer’s project, a large concrete test block was made with embedded targets to model the subsurface defects at depths ranging from 25 to 127 mm (1 to 5 inches) in concrete. Data were collected for six months and were analyzed to determine the environmental conditions that would make detection of subsurface delaminations more possible during practical bridge inspections. This research trend continued until 2013, with Rilya Rumbayan’s work [13]. Rumbayan developed a numerical model to predict thermal contrasts in subsurface delamination based on a given set of data from Washer’s [12] research. These studies have just focused on the passive IRT technique when considering the impact of environment on the concrete detection. Therefore, in this study, an extensive experimental investigation was carried out on the size and depth of sub-surface delaminations with various environmental conditions focusing on the active IRT technique as mentioned above.

This paper is organized as follows. First, the fundamentals of infrared thermography are introduced in Section 3 with discussion on the appropriate input parameters for setting up the infrared (IR) camera. Section 4 contains the preparation of testing specimen, testing method, and instrumentation. The experimental results are discussed in Section 5, where the analysis focuses on the impact of meteorological factors on the testing results on both area and depth detection of delaminations. Finally, the conclusion is in Section 6.

## 3. Fundamentals of Infrared Thermography

**Figure 2:**
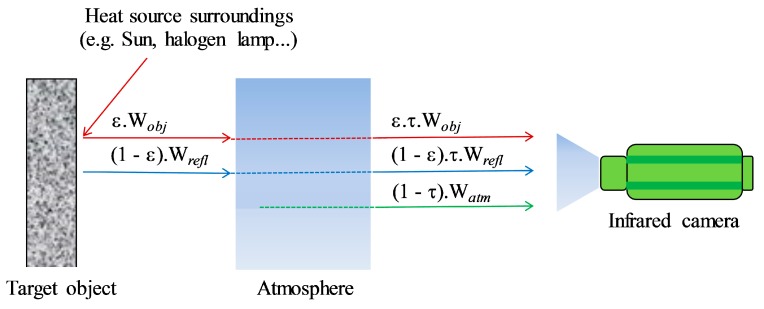
Principle of an infrared (IR) thermal camera receiving radiation.

IRT is known as a technique that measures the infrared radiation emitted by an object and converts the energy detected into a temperature value [14]. Each pixel then represents a thermal spot that is shown on a display or image, the so-called thermal infrared image. However, when measuring an object, the IR camera not only receives the radiation emitted from the object, but also the radiation from other sources such as surrounding objects or the atmosphere, as illustrated in Figure 2.

The total radiation (*W_tot_*) received by the IR camera comprises the emission of the object, ε·τ *W_obj_*, the emission of the surroundings and reflected by the object, (1 − ε)⋅τ⋅*W_refl_*, and the emission of the atmosphere, (1 − τ)·*W_atm_*, [14,15,16] as follows:

(1) $$\begin{array}{l} W_{tot}=\varepsilon \cdot \tau \cdot W_{obj}+\left(1-\varepsilon \right)\cdot \tau \cdot W_{refl}+\left(1-\tau \right)\cdot W_{atm}\\ =\varepsilon \cdot \tau \cdot \sigma \cdot T_{obj}^{4}+\left(1-\varepsilon \right)\cdot \tau \cdot \sigma \cdot T_{refl}^{4}+\left(1-\tau \right)\cdot \sigma \cdot T_{atm}^{4} \end{array},$$

The temperature of an object can be calculated from Equation (2), where σ is the Stefan-Boltzmann constant (=5.67 cm × 10^−8^ W/m^2^·K^4^). Other parameters must be set up in the camera, including the emissivity of the object material (ε), the transmittance of the atmosphere (τ), the reflected temperature (*T_refl_*), and the atmospheric/ambient temperature (*T_atm_*).

(2) $$T_{obj}=\sqrt[{4}]{\frac{W_{tot}-\left(1-\varepsilon \right)\cdot \tau \cdot \sigma \cdot T_{refl}^{4}-\left(1-\tau \right)\cdot \sigma \cdot T_{atm}^{4}}{\varepsilon \cdot \tau \cdot \sigma }\;},$$

According to Equation (2), three parameters are needed to automatically calculate the atmospheric transmission (τ) using the IR camera: survey distance, relative humidity, and atmospheric temperature. The formula used by the FLIR camera is as follows [17,18]:

(3) $$\tau \left(d,\omega \right)=K_{atm}\cdot \exp\left[-\sqrt{d}\left(\alpha_{1}+\beta_{1}\sqrt{\omega }\right)\right]+\left(1-K_{atm}\right)\cdot \exp\left[-\sqrt{d}\left(\alpha_{2}+\beta_{2}\sqrt{\omega }\right)\right],$$

(4) $$\omega \left(\omega_{\%},T_{atm}\right)=\omega_{\%}\cdot \exp\left(h_{1}+h_{2}\cdot T_{atm}+h_{3}\cdot T_{atm}^{2}+h_{4}\cdot T_{atm}^{3}\right),$$

Here, *ω* is the coefficient indicating the content of water vapor in the atmosphere and *ω*% is the relative humidity. Other parameters include distance (d), the scaling factor for the atmosphere damping (*K_atm_* = 1.9), attenuation for atmosphere without water vapor (*α*_1_, *α*_2_), attenuation for water vapor (*β*_1_, *β*_2_), and *h*_1_ = 1.5587, *h*_2_ = 6.939 × 10^−2^, *h*_3_ = −2.7816 × 10^−4^, and *h*_4_ = 6.8455 cm × 10^−7^. 

**Figure 3:**
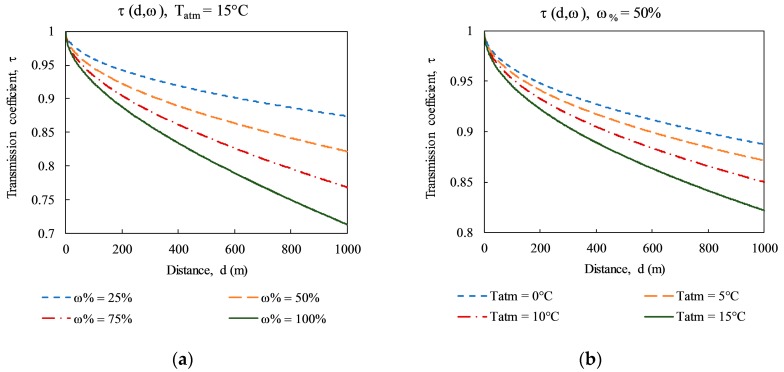
Characteristics of atmospheric transmission coefficient τ = *f*(d) for longwave band LW ThermaCAM PM 595 camera: (**a**) Function of the relative humidity *ω*%; (**b**) function of the atmospheric temperature *T_atm_* [17].

The characteristics were specified using Equations (3) and (4) by Minkina et al. [17], and shown in Figure 3 with specific parameters of *α*_1_ = 0.0066, *α*_2_ = 0.0126, *β*_1_ = −0.0023, and *β*_2_ = −0.0067.

As shown in Figure 3, the atmospheric transmission (τ) is close to 1.0 when the survey distance is fairly small (e.g., a common distance for active IRT is from approximately 3 to 5 m). Therefore, only the emissivity (ε) and the reflected temperature (*T_refl_*) in Equation 2 are necessary for measuring the target object under a survey at a near distance. Furthermore, the emissivity of a dry concrete surface has been shown to be a stable value of 0.95 through many studies, and is commonly specified in textbooks, camera user manuals [15,16,19], and by the American Concrete Institute (ACI) for infrared thermography methods [20]. In addition, an aluminum foil was used to evaluate the emissivity of the concrete specimen, and the result was very close to 0.95. Therefore, in this study, the emissivity is fixed at a value of 0.95 throughout all tests. The reflected temperature is the same as the atmospheric temperature for measuring an object with high emissivity in most cases [16]. Hence, for the IR camera set-up, it is considered that the reflected temperature corresponds to the ambient temperature.

## 4. Experimental Design and Procedure

### 4.1. Preparation of Testing Specimen

**Figure 4:**
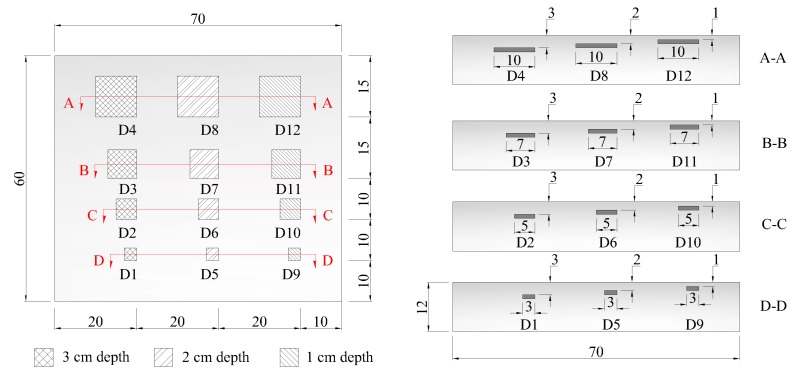
Concrete specimen with embedded artificial delaminations.

To assess the influence of environmental conditions on the subsurface delamination detection, a concrete specimen was fabricated with the design compression strength of 28 MPa. The concrete mixture ratio of water, cement, sand, and aggregate are 0.5, 1.0, 1.2, and 2.6, respectively. The thermal diffusivity (α) of the concrete specimen is estimated to be 1.636 cm^2^/min as described below. Twelve delaminations were simulated with different dimensions and depths embedded inside the concrete specimen. The delaminations considered in this study are square shapes of polystyrene, denoted hereafter as a capital letter “D”, with the size of 10 cm × 10 cm, 7 cm × 7 cm, 5 cm × 5 cm, and 3 cm × 3 cm at 1 cm deep (D9 to D12), 2 cm deep (D5 to D8), and 3 cm deep (D1 to D4) from the specimen's surface as shown in Figure 4. The delaminations are simulated using polystyrene, the thermal conductivity of which is 0.027 W/m·°C, which is similar to that of air (0.024 W/m·°C [21]) and is thus expected to behave in a similar manner to that of an air void when receiving heat flux [22].

### 4.2. Testing Method

**Figure 5:**
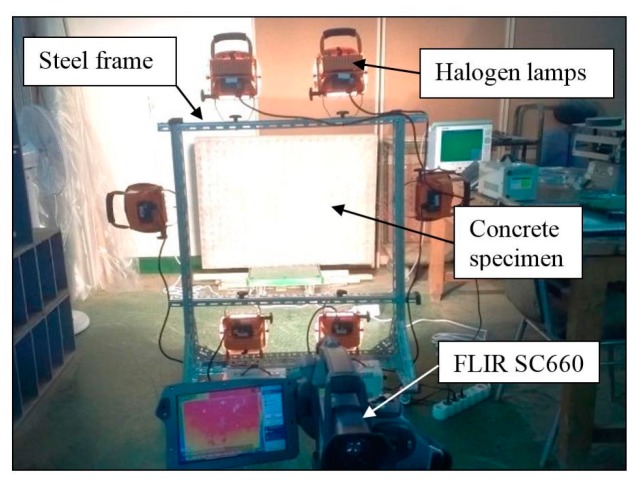
Photograph of the thermography system.

Twelve cases are considered in a series of experiments that are carried out in the laboratory on different days under different environmental conditions. Six halogen lamps (providing a maximum energy output of 3000 watts) as illustrated in Figure 5 were placed at 1.2 m from the specimen and were applied as an external heat source by providing heat flux to the concrete specimen. For this technique, the specimen was heated for 15 min and a long-wavelength IR thermal camera, FLIR SC660 [23], was used to record the variation in temperatures of the specimen every 10 s during both the heating and cooling periods. The major technical data of the camera are listed in Table 1. The camera was placed at 2.9 m from the specimen and was set at a constant emissivity value of 0.95 for all cases of testing. The ambient temperature was measured using an ambient temperature and humidity meter (CEM DT-615), ranging from 15.8 °C to 21.9 °C for the twelve cases, and the corresponding relative humidity was varied from 71% to 35%. Half of the experimental cases were tested on rainy days while the other half was tested on sunny days. The detailed values of the meteorological conditions for the IRT inspection are given in Table 2. The atmospheric transmission can be automatically calculated by the camera based on the information of camera distance, ambient temperature, and relative humidity, as mentioned previously.

## 5. Analysis of Experimental Results

In evaluating the defective depth and the percentage of delaminated areas in the concrete specimen, it is critical to consider the parameter of the maximum thermal contrast between the sound and defective areas, herein termed the “absolute contrast”. The thermal image clearly shows that as the absolute contrast increases, the detectability of the sub-surface defects increases. Therefore, this parameter is the main subject for analysis under the influence of atmospheric temperature and relative humidity, with the aim of taking a holistic view when applying this active thermographic method for concrete structures in the field.

### 5.1. Correlation between Ambient Temperature and Relative Humidity

**Figure 6:**
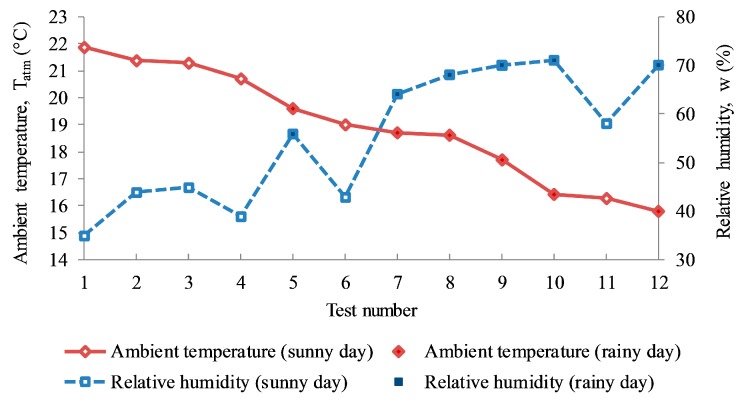
Correlation between ambient temperature and relative humidity.

The variation in the relative humidity occurred because the saturated vapor pressure is determined by the air temperature. As the water vapor content stays the same, the temperature increases, the relative humidity decreases substantially, and as the temperature drops, the relative humidity rises substantially [24]. The same behavior was shown for the twelve test cases in this study. When sorting all ambient temperature data in descending order, the general trend of relative humidity increased correspondingly, except for some test cases which showed low values of relative humidity, such as tests number 4 and 6 as shown in Figure 6.

The ambient temperature and relative humidity might influence the test results in different ways. For example, if the surrounding ambient temperature is higher than the temperature inside the concrete, heat flux will conduct into the concrete to achieve thermal equilibrium. The rate of heat transfer from the outside atmosphere to the concrete is controlled by convection [12]. However, since this study focuses on structures that are less affected by the wind speed, the heat transfer typically depends only on the heat conduction from the ambient air. In addition, the relative humidity of the atmosphere appears to influence the convection characteristics. It is expected that the heat flux would be transferred to the concrete more quickly in a humid atmosphere than in a dry atmosphere [25]. This leads to an increase of the thermal gradient between the outside surfaces and the core of the concrete. In this study, therefore, the absolute contrast in the defective area is separately investigated under the effects of ambient temperature and relative humidity in Section 5.2 and Section 5.3, respectively.

### 5.2. Effect of Ambient Temperature on the Absolute Contrast

From the experimental data, defective areas are easily identified as hotter than other surrounding sound areas because the delamination interrupts the heat transfer through the concrete. The area can be calculated by counting the number of pixels on the thermal images. As mentioned previously, as the thermal gradient increases, the defective boundaries become easier to identify. In this study, the temperature gradient, Δ*T*(*t*), (also termed the absolute contrast [6], or thermal contrast [26]) between the surface temperature of the defective area, *T_d_*(*t*), and that of the background sound area, *T_s_*(*t*), can be defined by the following equation [6],

(5) $$\Delta T\left(t\right)=T_{d}\left(t\right)-T_{s}\left(t\right),$$

**Figure 7:**
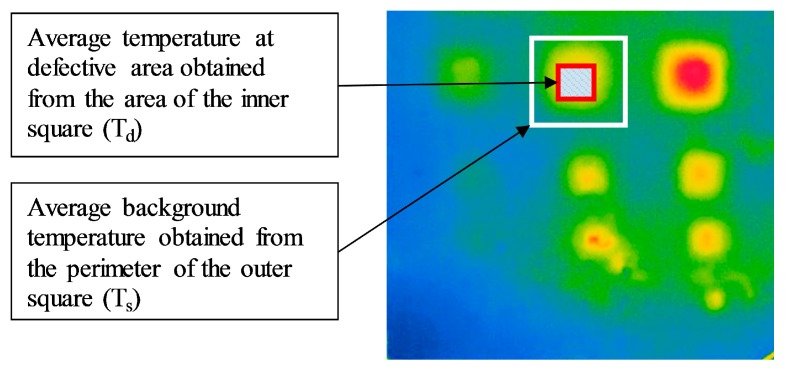
New evaluation method to calculate absolute contrast.

The absolute contrast was obtained by taking the average surface temperature within the boundaries of an area above the suspected defect minus the average temperature on the perimeter around the suspected defect as illustrated in Figure 7 [5]. The sizes of these two squares were changed according to the size of each delamination. This method has been shown to be the most effective as it diminishes the variability in selecting only one point [6,27] or a group of three points in the previous study [22].

In Washer et al.’s (2009) study on developing passive infrared thermography inspection technology for bridges, it was observed that when the rate of change of the ambient temperature was close to zero (i.e., the ambient temperature is constant), the absolute contrast in the testing concrete slab began to diminish. However, when receiving heat energy from the sun in the morning, the rapid change in the air temperature caused the development of the absolute contrast in the test block [12]. Corresponding to the active IRT technology applied on the concrete specimen in this study, similar outcomes were found to those Washer’s studies using passive thermography. As the ambient temperature increases, the absolute contrast on the defect also increases, and as the ambient temperature drops, the absolute contrast on the defect also decreases. On the other hand, it is generally recognized that the absolute contrast increases at a faster rate in the large and shallow delaminations than in the smaller and deeper delaminations.

**Figure 8:**
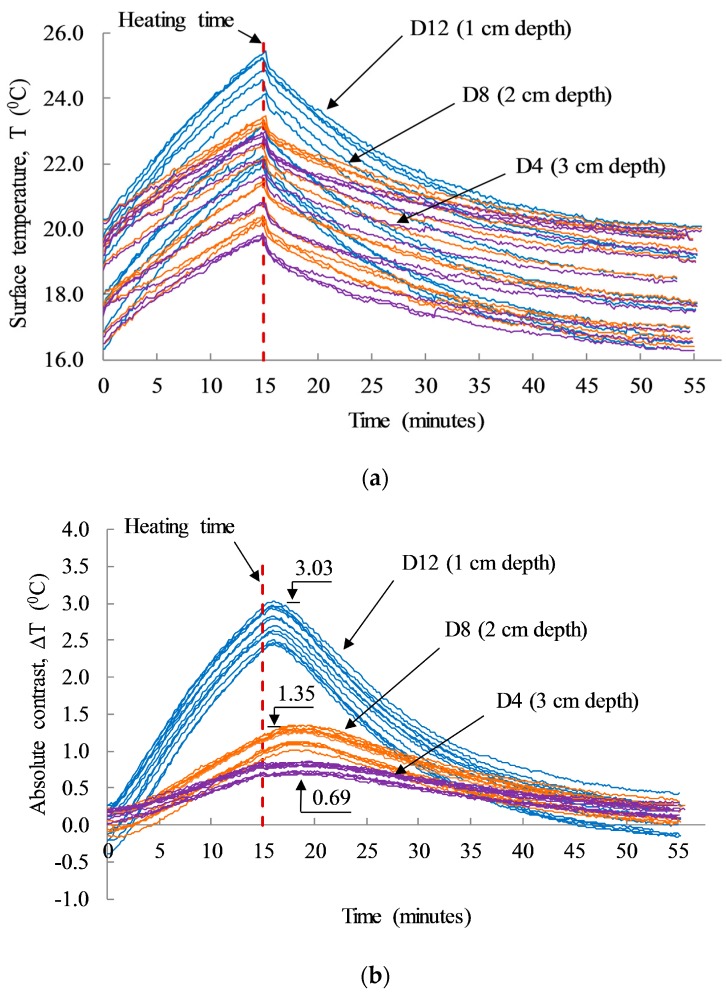
(**a**) Average surface temperature above the defects; and (**b**) average absolute contrast above the defects for a total of 55 min for both the heating and cooling time.

The results shown in Figure 8 illustrate the absolute contrasts obtained from delaminations D4, D8, and D12 having the same size of 10 cm × 10 cm and different depths under the twelve cases of dissimilar environmental conditions. The absolute contrast of the shallow delamination (D12) shows a higher temperature than the other delaminations. In addition, the group of twelve test cases displays different absolute contrasts for each delamination depth under varying atmospheric conditions. In particular, as the ambient temperature decreases from 21.9 °C to 15.8 °C, the absolute contrast of D12, D8, and D4 also decreased from 3.03 to 2.45, from 1.35 to 1.02, and from 0.86 to 0.69, respectively, as depicted in Figure 8b. This can be explained by considering that, when the ambient temperature decreases, the thermal gradient between the surfaces and core of the concrete also decreases. Consequently, the heat flux transferred from the heat sources (halogen lamps) to the concrete core decreased. Thus, the absolute contrast was similarly low under low ambient temperature with the same size and depth of defect, and the same heating time, type of heat source, and heat transfer energy.

**Figure 9:**
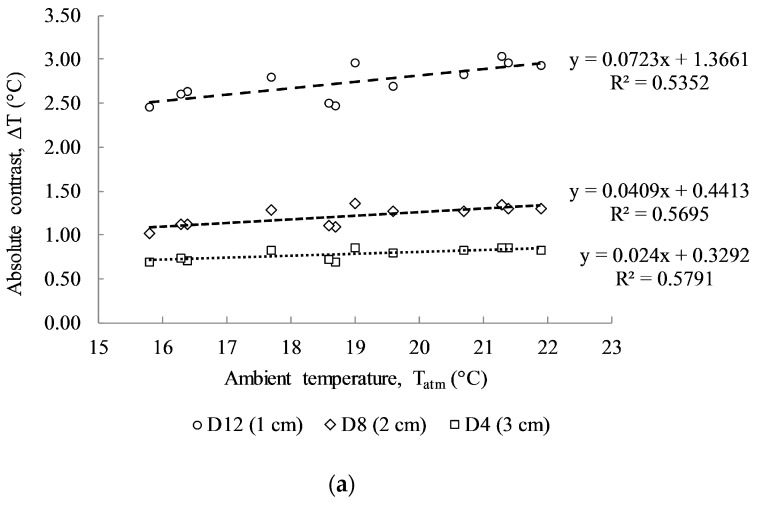

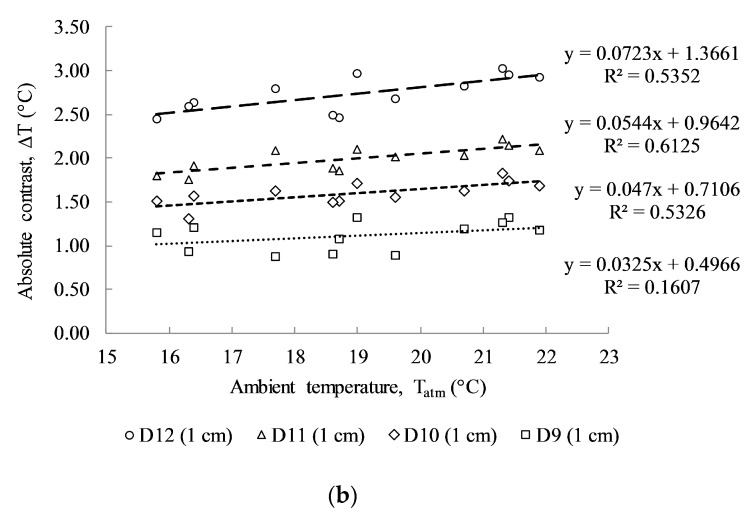
Relationship between absolute contrast and atmospheric temperature: (**a**) Same size delamination (10 cm × 10 cm) with different depths of delamination D12 (at 1 cm), D8 (at 2 cm) and D4 (at 3 cm); (**b**) same depth (1 cm) with different sizes of delamination D12 (10 cm × 10 cm), D11 (7 cm × 7 cm), D10 (5 cm × 5 cm), and D9 (3 cm × 3 cm).

Figure 9a illustrates the linear relationship between absolute contrast and ambient temperature, in which the correlation coefficient values (R^2^) give relatively low values of 0.5352, 0.5695, and 0.5791 corresponding to the delamination sizes of 10 cm × 10 cm at 1 cm, 2 cm, and 3 cm, respectively. The slopes of the results of the linear equations also indicate the positive correlation between the ambient temperature and the absolute contrast that was developed above the delaminations with different depths. Higher R^2^ values are obtained than those from the Washer’s field test (with R^2^ = 0.22). This might be due to the effects of wind speed, different solar loading, and cloud path during the test. In addition, the slope tendency of a linear line becomes steeper with shallower delamination. In particular, the slopes of linear regression lines are 0.0723, 0.0409, and 0.024 for delaminations D12, D8, and D4 at 1 cm, 2 cm and 3 cm depths, respectively. 

The trend of thermal development on the defective areas due to the increase of ambient temperature is also investigated for delaminations with different sizes, although with the same depth as shown in Figure 9b. Less steep slopes are obtained for smaller sizes of delamination; for example, the slope of linear regression is only 0.0325 for D9 (3 cm × 3 cm), whereas it is 0.047, 0.0544, and 0.0723 for delaminations D10 (5 cm × 5 cm), D11 (7 cm × 7 cm), and D12 (10 cm × 10 cm), respectively. It can be stated that the absolute contrasts are fairly low for defects with small size and deeper depth. Hence, the temperature gradient on the defect due to the change of ambient temperature is found to be difficult to assess.

### 5.3. Effect of Relative Humidity on the Absolute Contrast

The rate of convective heat transfer typically depends on the atmospheric temperature and wind speed [12], and is influenced by the relative humidity of the ambient air surrounding the concrete specimen [25]. In particular, a noticeable increase is observed in the convective heat transfer coefficient with an increase of the relative humidity. In other words, the effect on the detectability of the subsurface defect under a humid atmosphere might be greater than that under a dry atmosphere. 

**Figure 10:**
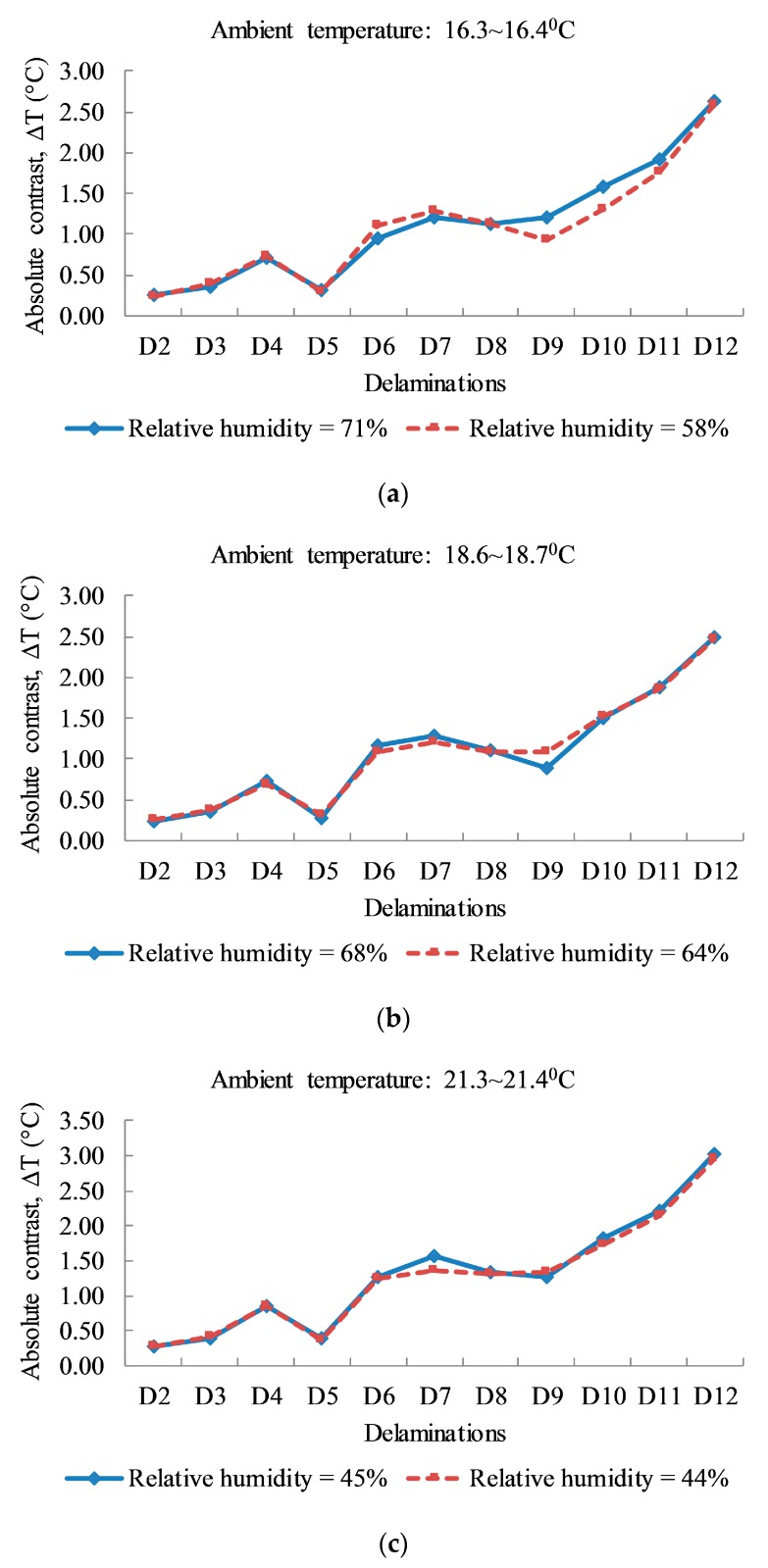
Relationship between absolute contrast and relative humidity: (**a**) for the ambient temperature from 16.3 °C to 16.4 °C; (**b**) for the ambient temperature from 18.6 °C to 18.7 °C; (**c**) for the ambient temperature from 21.3 °C to 21.4 °C.

In this study, to investigate the effect of relative humidity on the active thermographic method, data coinciding with ambient temperature (0.1 °C difference) with varying relative humidity are considered. These data are divided into three groups with the average atmospheric temperatures of 16.35 °C, 18.65 °C, and 21.35 °C, along with three pairs of relative humidity (71% and 58%), (68% and 64%), and (45% and 44%), respectively. All data are plotted in Figure 10, illustrating the change of absolute contrast under similar testing conditions, though with varying humidity. The difference of absolute contrast is more clearly shown in Figure 10a than in Figure 10b,c, especially for shallower delaminations from D9 to D12. The absolute contrast values when tested with high humidity show larger values than those tested in the low humid atmosphere. This might be due to the significant relative humidity difference shown in Figure 10a of approximately 13%, compared to 4% and 1% for other two cases shown in Figure 10b,c, respectively. For deeper delaminations from D2 to D8, the change of absolute contrasts due to the influence of humidity observed from this experiment is not significant. It should be noted that this judgment is based on limited data and that uncertainties occurred in the environmental conditions during testing. It is recommended that relative humidity needs to be considered as a secondary factor in a practical point of view when using active thermography in a field application.

In addition, while larger delaminations are normally more expected for higher temperatures than smaller temperatures at a similar depth, delamination D7 indicates unusual behavior. Particularly, the test results shown in Figure 10 indicate that the absolute contrast of delamination D7 (7 cm × 7 cm) has a higher temperature value than that of the larger delamination, D8 (10 cm × 10 cm) at the same depth of 2 cm. This could be due to non-uniform heating on the specimen surface and/or due to the reflection of the heat from the concrete surface, as reported by Vaghefi [5]. It is also observed in this research that the existence of tiny air voids in the concrete cover above both defects of D6 and D7 are due to the low compaction when fabricating the specimen. This leads to several hotter points above the defect location that might increase the average temperature of the defective area. It is noted that the selection of a suitable defective zone plays an important role in absolute contrast analysis. 

It is also important to note that absolute contrast values are significantly dependent on the method by which they are obtained. In the selection of the reference background temperature using the one- or three-point method as mentioned previously, the absolute contrast can easily vary if the reference point is moved. To deal with this problem, in this research the reference background temperature is obtained by taking the average of all pixels on the perimeter of the square around the defect; using this approach, it is expected that mistakes in selecting the background temperature can be avoided, as shown in Figure 7.

### 5.4. Depth Detection Under Environmental Effect

**Figure 11:**
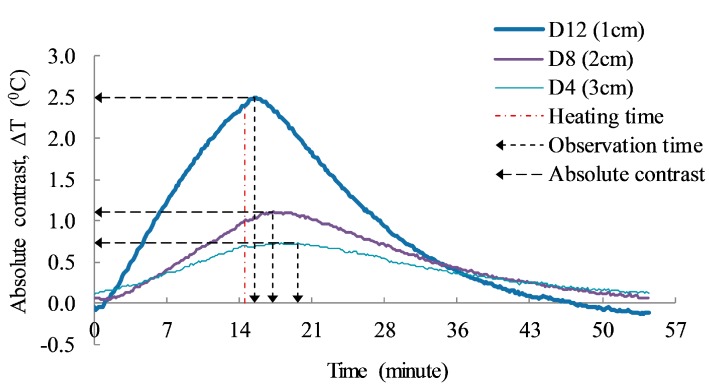
Typical graph for relationship of the observation time and absolute contrast.

The depth of defect has been quantified through the observation time (t) shown as the vertical dashed arrows in Figure 11. It is defined as the time when the absolute contrast remains constant or increases to the maximum contrast after the heating time [5,6].

The relationship between the observation time and the defective depth (in the first approximation) can be expressed as [6,12]

(6) $$z\approx {\left(t\cdot \alpha \right)}^{1/2},$$

and the thermal diffusivity can be defined as

(7) $$\alpha =\frac{K}{\rho \cdot C},$$

where specific heat capacity (*C*) refers to a measure of the ability of materials to absorb heat; thermal conductivity (*K*) is the measure of the heat transfer through materials, and ρ is the material density of the target object.

**Figure 12:**
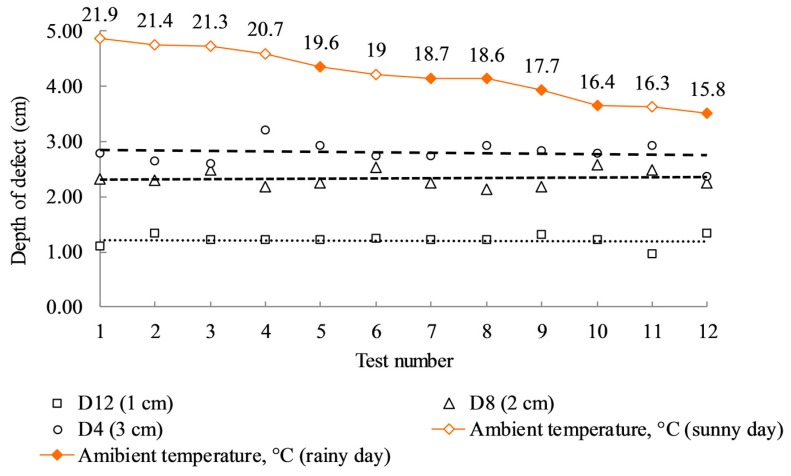
Relationship between depth of defects and ambient temperature.

Figure 12 illustrates the estimated defective depth values for delaminations D4, D8, and D12, sorted in the descending order of ambient temperature. The result shows that the estimated depth values fluctuate with different environmental conditions, and the fluctuation is relatively high for deep defects. However, the overall trend is maintained, despite a decline of ambient temperature. It can be inferred that the depth detection might be less affected by the environmental conditions such as ambient temperature and relative humidity, especially for shallow defects.

**Figure 13:**
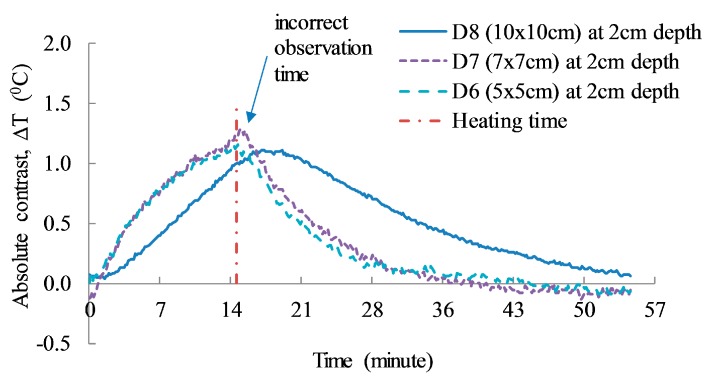
Absolute contrast versus time for delaminations D8, D7, and D6 at similar depths (case 8).

In this test, the delaminations D6 and D7 show the maximum contrast immediately after the 15-min heating time, indicating that the observation time is considerably less than 1 min. Thus, for these delaminations, the lack of information for depth detection caused incorrect estimation, as depicted in Figure 13. A number of very small air voids that exist above the delaminations could have resulted in faster thermal dissipation, leading to a reduction or even a loss of observation time.

**Figure 14:**
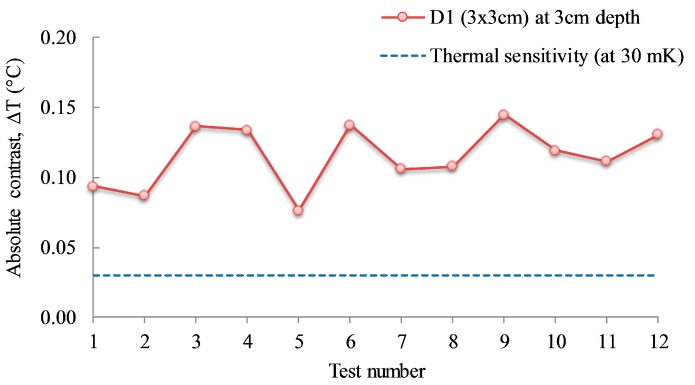
Absolute contrast of defect with width-to-depth ratio of 1.0.

For small and deep defects, which normally show low absolute contrast (e.g., lower than 0.5 °C according to the American Society for Testing and Materials (ASTM) requirement for field testing [28]), it was shown that the temperature values with time contain noises and the thermal curves are not clear enough to estimate the observation time [22]. Therefore, while the depth might have been detected incorrectly, the defects on the thermal image are still visible, except for the delamination with a ratio of width-to-depth of equal to or lower than 1.0 [5,11,22]. The absolute contrast value of delamination D1 (3 cm × 3 cm at 3 cm deep) with the width-to-depth ratio of 1.0 is lower than 0.15 °C for all cases of testing, which is impossible to visualize with the naked eye. However, the defect might be detectable if a high-quality IR camera having high resolution and thermal sensitivity/Noise Equivalent Temperature Difference (NETD) is used, and the tests are conducted under a less uncertain environment (e.g., an indoor test with virtually no wind effect). As shown in Figure 14, the average absolute contrast for the twelve cases of D1 is around 0.12 °C; this is measured by the FLIR SC660 camera because most of the values are higher than the thermal sensitivity of the camera (30 mK or 0.03 °C) [29]. Although visually undetectable, the delamination with the width-to-depth ratio of 1.0 might be detected by an image processing method with the correct selection of background temperature.

Thermal diffusivity must be acknowledged as an important parameter influencing the depth estimates, such as an observation time. Unlike metal materials, the density of concrete relates to air voids [30], which can lead to a change in thermal diffusivity. As seen in Equation (7), a lower material density can cause a higher diffusivity, thus a shorter observation time of the defect. In other words, the estimated defective depth will be less than the actual depth. Many researchers [10,11,31] have found that the thermal diffusivity of the material relates to the time of thermal wave propagation. Unfortunately, the results for concrete differ somewhat depending on the material density, type of aggregate, and the methods used to obtain this parameter. Moreover, the diffusion process weakens the heat as it moves deeper into the material [5]. Therefore, the diffusivity is difficult to estimate for concrete. The reference values obtained from several previous works are summarized in Table 3.

In this research, the concrete specimen was made of gneiss and granite aggregates and designed with a mixture ratio of water, cement, sand, and aggregate components as 0.5, 1.0, 1.2, and 2.6, respectively. The sizes of fine aggregates range from 4.75 to 12.5 mm (approximately 60%), whereas those of coarse aggregates range from 12.5 to 25 mm (approximately 40%).

**Figure 15:**
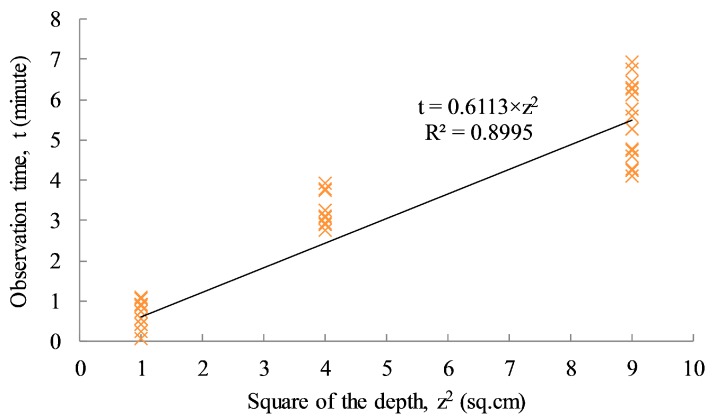
Observation time vs square of actual defective depth.

To estimate the thermal diffusivity of the concrete specimen, back-analysis from the linear relationship between the actual depth of each delamination and the observation time was carried out as plotted in Figure 15. Missing data for the over-heating and depth information are ignored in the analysis. The linear regression line with a high correlation coefficient value (R^2^ = 0.8995) indicates the reliability of the data set. The diffusivity of the concrete specimen can be estimated approximately by inverting the slope of the equation, α = 1/0.6113 = 1.636 cm^2^/min. This value can be applied to estimate the defective depth for the actual concrete structures in the field, having the same material characteristics.

In addition, the loss of contrast (*c*) is investigated to confirm the correction of its relationship against the cube of the depth as mentioned by Maldague [11]

(8) $$c\approx \frac{1}{z^{3}},$$

**Figure 16:**
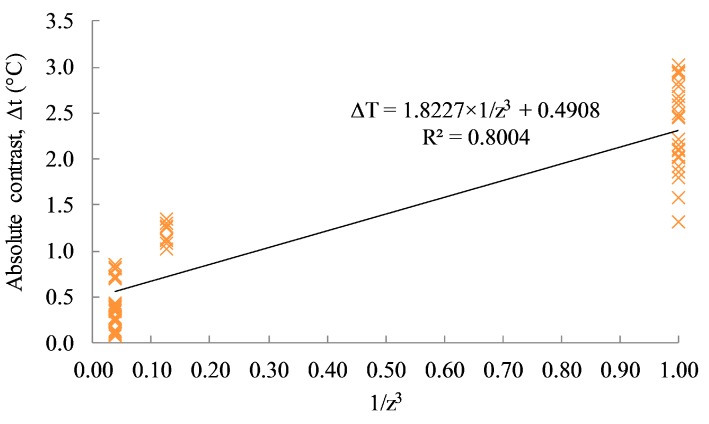
Absolute contrast vs. 1/*z*^3^.

The absolute contrast values versus the invert of the cubic depth (1/*z*^3^) of each delamination are also plotted in Figure 16. Based on the figure, the relationship between the absolute contrast and the inverse of the cubic depth (1/*z*^3^) is relatively accurate with a linear correlation coefficient value (R^2^) of 0.8004. It should be noted that the data are limited for only three different depths, four different sizes, and twelve cases of dissimilar environmental conditions.

This type of test should be carried out as many times as possible to obtain a large dataset relating to absolute contrast versus defective depth with a given external heating system (i.e., similar type of heat source, heating time, distribution layout, and distance of heat source). Then, similar to the process for the field test, with known environmental conditions, size of detect (observed from thermal image or camera screen), absolute contrast above defective area, and thermal characteristics of concrete, the depth of defect can be interpolated via the relationship obtained from the known data set without the need for the time-consuming task of analyzing the cooling curve to obtain the observation time for each delamination.

## 6. Conclusions

Active thermography test data obtained from a laboratory experiment were analyzed under different environmental conditions. The research focuses on the effects of different ambient temperatures and relative humidity conditions on the defective depth detection and the absolute contrast between the defective and non-defective areas of delaminations. Results of the experiment show that the absolute contrast between defective and sound areas increases with the increase of the ambient temperature and that the absolute contrast between defective and sound areas decreases with the decrease of the ambient temperature, and it increases at a faster rate with large and shallow delaminations than smaller and deeper delaminations.

The existence of a highly humid atmosphere might lead to an increase in the absolute contrast, especially for shallow delaminations of 1 cm deep. For deeper delaminations, the absolute contrast shows similar values for the two cases of the test at 71% and 58%, despite the difference in relative humidity of up to 13%. The results also indicate that, for shallow defects, the depth detection might be less affected by varying ambient temperature and relative humidity. Despite the deeper delaminations fluctuating under different environmental conditions, the overall trend indicates a leveling out with a decline of ambient temperature.

The thermal diffusivity of 1.636 cm^2^/min can be used for concrete structures in the field that have similar material characteristics as those used in this laboratory research. In addition, the loss of contrast versus cube of defective depth (1/*z*^3^) can be expressed through a linear relationship for practical purposes, in order to quickly estimate the defective depth based on a known data set, without the need for the time-consuming analysis of the cooling curve to obtain observation time for delaminations.

## Figures and Tables

**Table 1:** Technical data of FLIR camera SC660 [23].

Items	Parameters
IR resolution	640 × 480 pixels
Thermal sensitivity/NETD	<30 mK @ + 30 °C
Field of view (FOV)	24° × 18°/0.3 m
Spatial resolution (IFOV)	0.65 mrad
Focal Plane Array (FPA)	Uncooled microbolometer
Wavelength	7.5~13 μm
Temperature range	−40 °C~+120 °C
Accuracy	±1 °C or ±1% of reading

**Table 2:** Various meteorological conditions for the infrared thermography (IRT) Inspection.

Measurement No.	Meteorological Conditions	Ambient Temperature (°C)	Relative Humidity (%)
1	sunny day	21.9	35
2	sunny day	21.4	44
3	sunny day	21.3	45
4	sunny day	20.7	36
5	cloudy after the rain	19.6	66
6	sunny day	19.0	37
7	light rain	18.7	64
8	cloudy after the rain	18.6	68
9	showers	17.7	70
10	moderate rain	16.4	71
11	sunny day	16.3	58
12	light rain	15.8	70

**Table 3:** Values of thermal diffusivity of concrete.

Researcher	Density, ρ	Type of Aggregate	Thermal Diffusivity α = K/ρ C	Method
	(kg/m^3^)		(cm^2^/min)	
Maldague, 1993 [11]	2400	-	0.318	-
Maldague, 2001 [32]	2400 (dry)	-	0.318	-
	2500 (moist)	-	0.336	-
Lamond, 2006 [30]	-	Quartz	1.317	CRD-C 36, 37
		Quartzite	1.017	CRD-C 36, 37
		Limestone	0.917	CRD-C 36, 37
		Basalt	0.417	CRD-C 36, 37
		Expanded shale	0.250	CRD-C 36, 37
Vollmer, 2010 [19]	-	-	0.396 (at 20 °C)	-
Ahlborn, 2015 [6]	-	Quartz	1.425	IRT ^1^

Note: ^1^ Value taken by back analyzing the IRT on concrete slabs for defects with different sizes, shapes, and depths. The value of thermal diffusivity is estimated from the linear regression relationship between observation time and defect depth (R^2^ = 0.896).

## References

[1] Portland Cement Association (2002). Types and Causes of Concrete Deterioration.

[2] Bolleni K.N. (2009). Environmental Effects on Subsurface Defect Detection in Concrete Structures Using Infrared Thermography. Master’s Thesis.

[3] Maser K.R. Integration of Ground Penetrating Radar and Infrared Thermography for Bridge Deck Condition Evaluation. Proceedings of the NDTCE’09, Non-Destructive Testing in Civil Engineering Conference.

[4] Pollock D.G., Dupuis K.J., Lacour B., Olsen K.R. (2008). Detection of Voids in Prestressed Concrete Bridges Using Thermal Imaging and Ground-Penetrating Radar.

[5] Vaghefi K. (2013). Infrared Thermography Enhancements for Concrete Bridge Evaluation. Ph.D. Thesis.

[6] Ahlborn T.M., Brooks C.N. (2015). Evaluation of Bridge Decks Using Non-Destructive Evaluation at Near Highway Speeds for Effective Asset Management.

[7] Transportation Research Board (2013). Nondestructive Testing to Identify Concrete Bridge Deck Deterioration.

[8] IAEA (2002). Guidebook on Non-Destructive Testing of Concrete Structures.

[9] Clemena G.G., McKeel W.T. (1978). Detection of delamination in bridge decks with infrared thermography. Transp. Res. Rec..

[10] Cielo P., Haldague X., Deom A.A., Lewak R. (1987). Thermographic Nondestructive Evaluation of Industrial Materials and Structures. Mater. Eval..

[11] Maldague X.P.V. (1993). Nondestructive Evaluation of Materials by Infrared Thermography.

[12] Washer G.A., Fenwick R.G., Bolleni N.K. (2009). Development of Hand-Held Thermographic Inspection Technologies.

[13] Rumbayan R. (2013). Modeling of Environmental Effects on Thermal Detection of Subsurface Damage for Concrete Bridges. Ph.D. Thesis.

[14] Usamentiaga R., Venegas P., Guerediaga J., Vega L., Molleda J., Bulnes F.G. (2014). Infrared Thermography for Temperature measurement and non-Destructive Testing. Sensors.

[15] Holst G.C. (2000). Common Sense Approach to Thermal Imaging, Bellingham.

[16] FLIR System Inc (2012). The Ultimate Infrared Handbook for R&D Professionals.

[17] Waldemar M., Klecha D. Modeling of Atmospheric Transmission Coefficient in Infrared for Thermovision Measurements. Proceedings of the Sensor 2015 and IRS2 2015 AMA Conferences.

[18] FLIR (2001). Toolkit IC2 Dig 16: Developers Guide 1.01 for AGEMA 550, 570, ThermaCAM PM 5 cm × 5 and the ThermoVision Family, Version B, FLIR Publication Number: 557 344.

[19] Vollmer M., Mollmann K.P. (2010). Infrared Thermal Imaging-Fundamentals, Research and Applications.

[20] American Concrete Institute (2013). Report on Nondestructive Test Methods for Evaluation of Concrete in Structures.

[21] Dupuis K.J. (2008). Nondestructive Testing of Concrete Box Girder Bridges Using Thermal Imaging. Ph.D. Thesis.

[22] Huh J., Tran Q.H., Lee J., Han D., Yim S. (2016). Experimental Study on Detection of Deterioration in Concrete Using Infrared Thermography Technique. Adv. Mater. Sci. Eng..

[23] FLIR System Inc. (2014). SC660 Catalog, Technical Data of FLIR SC660 Infrared Camera.

[24] Allen R.G., Pereira L.S., Raes D., Smith M. (1998). Crop Evapotranspiration: Guidelines for Computing Crop Water Requirements-FAO Irrigation and drainage paper 56. FAO Rome.

[25] Zhang J., Gupta A., Baker J. (2007). Effect of Relative Humidity on the Prediction of Natural Convection Heat Transfer Coefficients. Heat Trans. Eng..

[26] Brown J.R. (2005). Infrared Thermography Inspection of Fiber-Reinforced Polymer Composites Bonded to Concrete. Ph.D. Thesis.

[27] Brown J.R., Hamilton H.R. (2007). Heating Methods and Detection Limits for Infrared Thermography Inspection of Fiber-Reinforced Polymer Composites. ACI Mater. J..

[28] ASTM D4788-07 (2007). Standard Test Method for Detecting Delaminations in Bridge Decks Using Infrared Thermography.

[29] FLIR Systems AB (2011). Thermal Imaging Guidebook for Industrial Applications.

[30] Lamond J.F., Pielert J.H. (2006). Significance of tests and Properties of Concrete and Concrete-Making Materials.

[31] Cielo P., Maldague X., Krapez J.-C., Lewak R., Bussiere J.F., Monchalin J.P., Ruud C.O., Green R.E. (1987). Optics-based techniques for the characterization of composites and ceramics. Nondestructive Characterization of Materials II.

[32] Maldague X.P. (2001). Theory and Practice of Infrared Technology for Nondestructive Testing.

